# Effectiveness and Safety of Two Thrombopoietin Receptor Agonists: A Retrospective Cohort Study of Adults With Primary Immune Thrombocytopenia

**DOI:** 10.7759/cureus.113083

**Published:** 2026-07-21

**Authors:** Diego G Lanza, Leonardo J Enciso, Luis G Moncaleano, María A Rodríguez

**Affiliations:** 1 Department of Internal Medicine, Universidad Nacional de Colombia, Bogota, COL; 2 Department of Medicine, Hospital Universitario Nacional de Colombia, Bogota, COL; 3 Department of Hematology, Hospital Universitario Nacional de Colombia, Bogota, COL

**Keywords:** adult, chronic idiopathic thrombocytopenia, eltrombopag, idiopathic, purpura, romiplostim, thrombocytopenic

## Abstract

Introduction: Evidence on the comparative effectiveness and safety of thrombopoietin receptor agonists (TPO-RAs) in adults with primary immune thrombocytopenia (ITP) is scarce. We conducted a retrospective cohort study at a Colombian university hospital to describe the effectiveness and safety of romiplostim (ROM) and eltrombopag (ELT) and to explore comparative outcomes between treatment groups.

Methodology: A retrospective cohort study was conducted using medical records of adults with primary ITP treated at the Hospital Universitario Nacional de Colombia (Colombia National University Hospital) between 2016 and 2023. Effectiveness and safety outcomes are described, and the probability of platelet response was determined using Kaplan-Meier analysis.

Results: A total of 58 patients were included (63.8% women), with a mean age of 48 years. The median number of prior treatments was 3.00 (interquartile range (IQR), 3.00-4.00) for the ROM group and 2.00 (IQR, 2.00-3.00) for the ELT group (p < 0.001). The median time to achievement of the first platelet response was 37 days (95% confidence interval (CI), 19-61) in patients treated with ELT and 71 days (95% CI, 12-162) in those treated with ROM (p = 0.50). The proportion of patients achieving at least one platelet response during follow-up was 97.3% for ELT and 100% for ROM (p = 1.000). No statistically significant difference in the probability of achieving a platelet response was observed between treatment groups (log-rank p = 0.461). Thrombotic events and treatment-related adverse events were infrequent.

Conclusions: In this real-world retrospective study, no statistically significant differences were observed between ELT and ROM in time to platelet response or the probability of platelet response. Safety outcomes were infrequent in both groups. Patients treated with ROM had received more prior therapies and more frequently had undergone splenectomy, suggesting a population with more advanced disease at treatment initiation. These findings provide comparative real-world data from a Latin American cohort and should be interpreted as exploratory and hypothesis-generating.

## Introduction

Primary immune thrombocytopenia (ITP) is a disease characterized by immune system-mediated platelet destruction and decreased platelet production. Although the exact pathophysiological mechanisms involved are not fully understood, proposed mechanisms include the development of autoantibodies directed against platelet antigens resulting in their destruction in the reticuloendothelial system, as well as damage to megakaryocytes in the bone marrow and impaired platelet production [1,2]. Primary ITP is estimated to have an annual incidence of approximately 3/100,000 people, with peaks observed in young women and older adults [3]. Primary ITP is classified as newly diagnosed when it lasts less than three months, persistent when it lasts between three months and one year, and chronic when it lasts more than one year [4].

First-line treatment is based on the use of glucocorticoids, such as prednisone or high-dose dexamethasone, as well as intravenous immunoglobulin G if a rapid increase in platelet count is required [5]. However, these measures are limited by the multiple adverse effects of prolonged steroid treatment, as well as the short duration of action and high cost of intravenous immunoglobulin G [2]. In the second-line setting, medications such as rituximab are available; however, these carry risks associated with immunosuppression, as well as evidence of a lower durable response compared to thrombopoietin receptor agonists (TPO-RAs; 60% for TPO-RAs vs. 39% for rituximab) [5,6]. In 2018, the United States Food and Drug Administration (FDA) approved fostamatinib, a splenic tyrosine kinase inhibitor that targets the inhibition of platelet phagocytosis in the reticuloendothelial system and has a durable platelet response of approximately 18% but with a lower risk of immunosuppression and faster platelet responses compared to rituximab [6]. Other drugs that are used, such as azathioprine, mycophenolate mofetil, cyclosporine, decitabine, and cyclophosphamide, have low-quality evidence and are associated with higher risks of immunosuppression or carcinogenesis [6].

The development of TPO-RAs as second-line drugs has led to a greater response to treatment and a reduction in the need for splenectomy or immunosuppression [5]. Currently, the 2019 American Society of Hematology guidelines recommend the use of TPO-RAs, either eltrombopag (ELT) or romiplostim (ROM), for the treatment of patients with persistent or chronic ITP resistant to corticosteroid therapy with a very low level of evidence [5]. These medications are recommended as the second-line treatment of choice for patients with chronic ITP refractory to first-line therapies and even for the management of newly diagnosed primary ITP, according to expert consensus [7].

ROM is a synthetic protein composed of four peptides linked to a constant Fc fragment of immunoglobulin G. This drug binds to the extracellular region of the thrombopoietin receptor, generating a signaling cascade through the JAK/STAT and MAPK pathways, stimulating platelet production [8]. It was approved by the FDA in 2008 based on two phase III placebo-controlled studies that demonstrated a sustained platelet response (platelets >50,000/μL for at least six of the final eight weeks) in 38% of splenectomized and 61% of non-splenectomized patients in the ROM group, compared with 5% of splenectomized and 0% of non-splenectomized patients in the placebo group [9]. These findings were corroborated in a long-term, open-label study with 292 patients, in which 94.5% achieved a platelet response >50,000/μL [10]. ELT is a small, non-peptide molecule that binds to the transmembrane region of the thrombopoietin receptor, stimulating platelet production through the same signaling cascade as ROM. It was approved by the FDA in 2008 after two randomized controlled clinical trials and one long-term open-label study [11]. The RAISE study was a phase III study with 197 patients who received ELT 50 mg daily vs. placebo with a platelet response achieved in 79% of patients treated with ELT compared with 28% of the placebo group during the six months of treatment [11]. In the EXTEND study of 300 patients who had previously participated in a study with ELT, 89% achieved platelet counts >50,000/μL during treatment [12].

Although studies have demonstrated the efficacy and safety of TPO-RAs in adults [6,8], including some meta-analyses of indirect comparisons [13,14], head-to-head comparisons between these drugs are lacking. Consequently, comparative evidence for outcomes such as time to platelet response, probability of platelet response, clinically significant bleeding events, or thrombotic events remains limited [7].

Furthermore, differences in clinical response to TPO-RAs have been postulated at both the individual and population levels [15], suggesting the possibility of switching between different TPO-RAs when an adequate platelet response is not achieved, with documented satisfactory results [15,16]. Genetic variations in the enzymes and intermediates responsible for the metabolism of each molecule have been proposed [15]. Studies in the Latin American population have been very scarce. The pivotal efficacy and safety study for ELT (RAISE) [11] included 23 countries globally, with Peru being the only Latin American participant. The pivotal study for ROM included only patients from the United States and Europe [9].

Given the lack of head-to-head comparative studies between ROM and ELT [7], the previously reported geographic variations in ITP populations and treatment practices [2], and the limited representation of Latin American patients in pivotal clinical trials [9,11], we conducted this retrospective cohort study using real-world data to describe the effectiveness and safety of ROM and ELT in adults with primary ITP treated at a Colombian university hospital and to explore comparative outcomes between treatment groups.

## Materials and methods

We present a real-world retrospective cohort study conducted at a teaching hospital in Bogotá, Colombia. A review of electronic health records was performed for all adults diagnosed with primary ITP and treated with ROM or ELT at the Hospital Universitario Nacional de Colombia (Colombia National University Hospital) between January 2016 and June 2023. The study was approved by the ethics committee of the hospital; the approval is recorded in the minutes of the meeting No. CEI-HUN-ACTA-2023-12 (Project ID: CEI-2023-10-02). 

Patient selection

Electronic health records were filtered to select patients according to ICD-10 classification [17] as D693 Idiopathic Thrombocytopenic Purpura, D694 Other Primary Thrombocytopenias, or D473 Thrombocytopenia (Essential Hemorrhage), with further review to include only adult patients with primary ITP and treatment with either ROM or ELT between January 1, 2016, and June 30, 2023.

Exclusion criteria included secondary ITP, aplastic anemia, and hereditary thrombocytopenias or congenital bone marrow failure syndromes.

Data extraction was performed using a specific data collection form in the REDCap platform [18]. Data were extracted independently with revision by two authors, and any disagreement was resolved by consensus or with the help of a third author.

The study exposure was defined as treatment with either ELT or ROM. Follow-up was defined as the interval from initiation of TPO-RA therapy to the date of the last available patient visit recorded in the medical record. TPO-RA doses were initiated and adjusted at the discretion of the treating hematologist according to platelet response and institutional practice, which was based on international ITP guidelines.

Effectiveness outcomes included time to the first platelet response; platelet response and complete platelet response at three, six, and 12 months after treatment initiation; therapeutic failure; and steroid dependence. Platelet response, complete platelet response, therapeutic failure, and steroid dependence were defined according to the standardized criteria proposed by Rodeghiero et al. [4]. The number of patients who discontinued treatment because of remission (platelet count >100,000/µL for >1 year) was also recorded for each TPO-RA.

Regarding treatment safety, thrombotic events and adverse events, including hepatotoxicity and myelofibrosis, were evaluated. The number of major bleeding events, defined by the World Health Organization (WHO) grading system for bleeding [19] as WHO grades 3-4, and clinically significant bleeding events (WHO grade ≥2) was assessed.

Statistical analysis

A descriptive statistical analysis was performed, including medians, interquartile ranges (IQRs), and percentages. Since the data obtained showed a non-normal distribution, non-parametric methods were used for their analysis, with differences in categorical variables analyzed using the Fisher exact test and differences in continuous variables using the Mann-Whitney U test.

Kaplan-Meier curves were constructed for the platelet response probability survival analysis, with calculation of the log-rank test. Time zero for the Kaplan-Meier analysis was defined as the date of initiation of TPO-RA therapy. The event was the achievement of platelet response according to the predefined study criteria. Patients with missing dates of platelet response were excluded from the time-to-event analysis because the event time could not be determined. Patients who did not achieve a platelet response were treated as censored at their last available follow-up. 

Missing data were not imputed. For the analyses of platelet response at three, six, and 12 months, patients with unavailable response data were retained as an "Unknown" category in the contingency tables. Given the retrospective, descriptive nature of the study, no adjustment for multiple comparisons was performed; therefore, all comparative analyses should be considered exploratory and hypothesis-generating. No a priori sample size or statistical power calculation was performed because all eligible patients meeting the inclusion criteria during the study period were included. Statistical analyses were performed using R software version 4.5.2 [20].

## Results

A total of 112 patients who met the inclusion criteria were identified. Fifty-four patients were excluded for the following reasons: 14 due to insufficient data, 33 with a follow-up time of less than three months, two who received treatment outside the follow-up period, and five due to concomitant treatment with ROM and ELT. Among the 58 included patients, 63.8% were women and the mean age was 48 years. Fewer than half had prior comorbidities (Tables 1, 2).

**Table 1:** Population comorbidities. *Charlson comorbidity index: a weighted comorbidity score used to predict mortality [21]. †No events of leukemia, lymphoma, HIV infection, metastatic tumor, hemiplegia, chronic obstructive pulmonary disease, dementia, chronic kidney disease, mild liver disease (hepatitis or cirrhosis without portal hypertension), or severe liver disease (cirrhosis with portal hypertension) were reported.

Variable	Population n = 58
Smoking, n (%)	9 (15.5)
Hypertension, n (%)	14 (24.1)
Diabetes, n (%)	9 (15.5)
Dyslipidemia, n (%)	6 (10.3)
Obesity, n (%)	3 (5.2)
Alcoholism, n (%)	1 (1.7)
Fatty liver disease, n (%)	2 (3.4)
Heart failure, n (%)	3 (5.2)
Peripheral artery disease, n (%)	1 (1.7)
Connective tissue disease, n (%)	2 (3.4)
Peptic ulcer disease, n (%)	2 (3.4)
Localized tumor, n (%)	4 (6.9)
Charlson, n (%) *^,^^ †^	
0	31 (53.4)
1–2	11 (19)
3–6	15 (25.8)
>7	1 (1.7)

**Table 2:** General characteristics of the study population. *SD: Standard deviation; TPO-RA: thrombopoietin receptor agonist

Variable	Population n = 58
Mean age (SD)*	48.91 (17.27)
Women n(%)	37 (63.8)
Previous thrombosis n(%)	2 (3.4)
Thrombophilia n(%)	0 (0)
Anticoagulant use n(%)	4 (6.9)
Initial platelet count, median (IQR)	28000 (15250, 32000)
TPO-RA initiation in chronic phase n(%)	44 (75.9)
Previous therapies, mean (SD)*	2.57 (1.08)
Previous splenectomy, n(%)	14 (24.1)

The median platelet count at treatment baseline with a TPO-RA was 28,000/µL (IQR, 15250-32000/µL). A large proportion of patients (75.9%) were in the chronic phase of the disease; most had already received two other treatment modalities, and less than 25% had undergone a previous splenectomy.

The characteristics of the population stratified by TPO-RA showed no statistically significant differences between the two groups (Tables 3, 4). The only exceptions were the median number of previous therapies, which was 3.00 (IQR, 3.00-4.00) in the ROM group and 2.00 (IQR, 2.00-3.00) in the ELT group (p < 0.001), and the proportion of patients who had previously undergone splenectomy, which was 13.0% in the ELT group and 42.9% in the ROM group (p = 0.0232). 

**Table 3:** Characteristics by TPO-RA type. IQR: interquartile range; SD: standard deviation; Inf: infinite; NA: not applicable; OR: odds ratio; W: Mann-Whitney W statistic; TPO-RA: thrombopoietin receptor agonist

Variable	Eltrombopag	Romiplostim	p-value	Test	Value
n	37	21			
Age median (IQR)	42.00 (35.00, 64.00)	47.00 (39.00, 61.00)	0.565	Mann - Whitney (W)	353
Women (%)	25 (67.6)	12 (57.1)	0.571	Fisher exact (OR)	0.645
Previous thrombosis (%)	0 (0.0)	2 (9.5)	0.127	Fisher exact (OR)	Inf.
Thrombophilia (%)	0 (0.0)	0 (0.0)	NA		
Anticoagulant use (%)	1 (2.7)	3 (14.3)	0.130	Fisher exact (OR)	5.81
Initial platelet count, median (IQR)	29000 (19000, 42000)	20000 (14000, 32000)	0.250	Mann - Whitney (W)	691
TPO-RA initiation in chronic phase (%)	26 (70.3)	18 (85.7)	0.220	Fisher exact (OR)	2.5
Previous therapies, median (IQR)	2.00 (2.00, 3.00)	3.00 (3.00, 4.00)	<0.001	Mann - Whitney (W)	175
Previous splenectomy (%)	5 (13.5)	9 (42.9)	0.0232	Fisher exact (OR)	4.65
Median duration of follow-up (IQR), days	716.0 (287.0–1186)	955.0 (567.0–1451)	0.304	Mann - Whitney (W)	555

**Table 4:** Comorbidities by TPO-RA type. *No events of leukemia, lymphoma, HIV infection, metastatic tumor, hemiplegia, chronic obstructive pulmonary disease, dementia, chronic kidney disease, mild liver disease (hepatitis or cirrhosis without portal hypertension), or severe liver disease (cirrhosis with portal hypertension) were reported. Inf: Infinite; OR: odds ratio; W: Mann-Whitney W statistic; TPO-RA: thrombopoietin receptor agonist.

Variable	Eltrombopag	Romiplostim	p-value	Test	Value
Smoking (%)	5 (13.5)	4 (19.0)	0.710	Fisher exact (OR)	1.49
Hypertension (%)	6 (16.2)	8 (38.1)	0.108	Fisher exact (OR)	3.11
Diabetes (%)	4 (10.8)	5 (23.8)	0.262	Fisher exact (OR)	0.388
Dyslipidemia (%)	4 (10.8)	2 (9.5)	1.000	Fisher exact (OR)	0.870
Obesity (%)	1 (2.7)	2 (9.5)	0.547	Fisher exact (OR)	3.7
Alcoholism (%)	0 (0.0)	1 (4.8)	0.362	Fisher exact (OR)	Inf
Fatty liver disease (%)	1 (2.7)	1 (4.8)	1.000	Fisher exact (OR)	1.78
Heart Failure (%)	1 (2.7)	2 (9.5)	0.547	Fisher exact (OR)	3.7
Peripheral artery disease (%)	1 (2.7)	0 (0.0)	1.000	Fisher exact (OR)	0
Connective tissue disease (%)	0 (0.0)	2 (9.5)	0.127	Fisher exact (OR)	Inf
Peptic ulcer disease (%)	1 (2.7)	1 (4.8)	1.000	Fisher exact (OR)	1.78
Localized tumor (%)	4 (10.8)	0 (0.0)	0.286	Fisher exact (OR)	0
Charlson (%)*			0.798	Mann - Whitney (W)	583.5
>7	0 (0.0)	1 (4.8)			
0	20 (54.1)	11 (52.4)			
1 - 2	7 (18.9)	4 (19.1)			
3 - 6	10 (27)	5 (23.8)			

**Figure 1:**
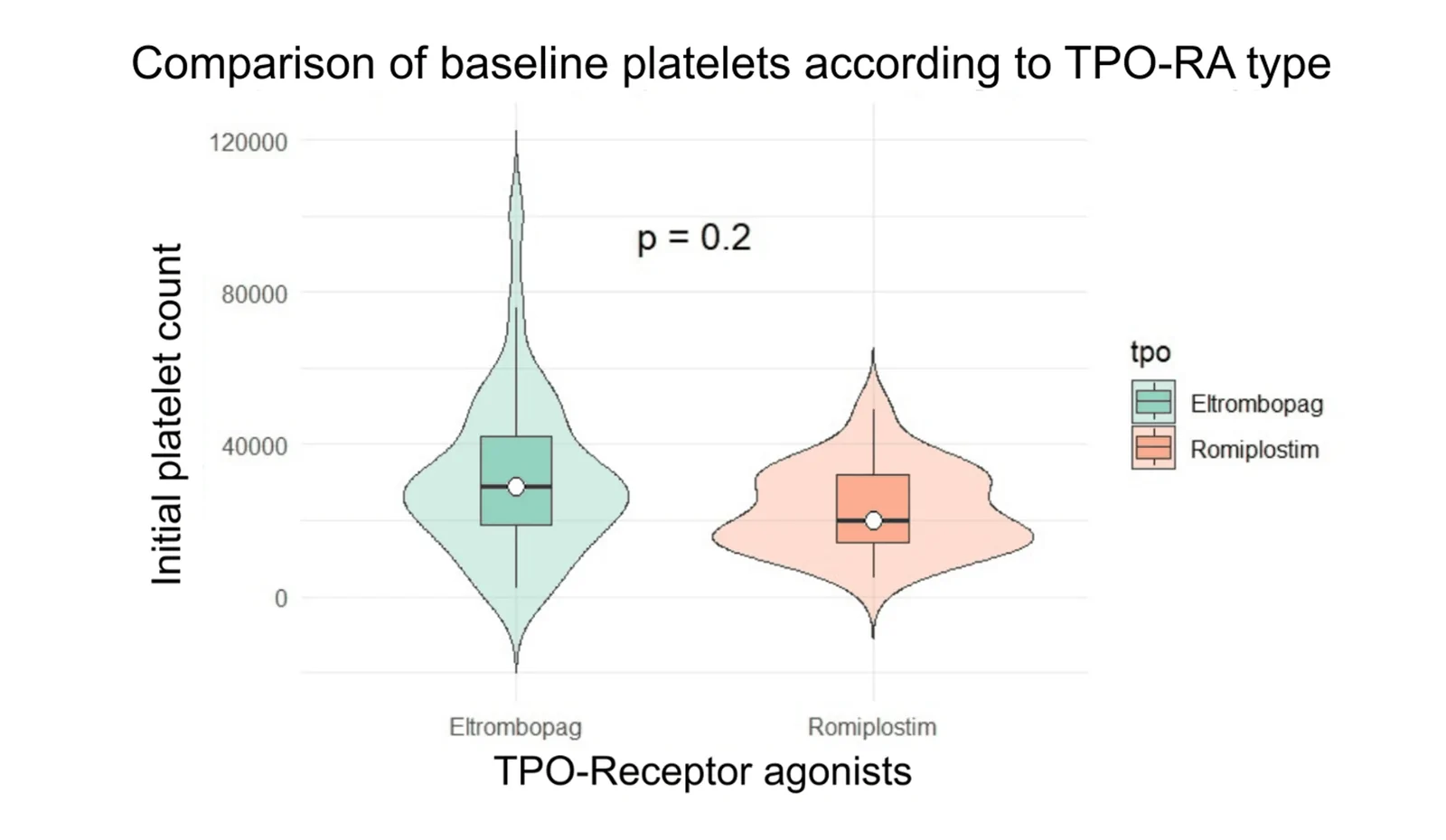
Violin plot comparing baseline platelets according to the TPO-RA. TPO: thrombopoietin; TPO-RA: thrombopoietin receptor agonist. The ROM group shows a bimodal distribution. Figure generated using R software [20]. TPO-RA: Thrombopoietin receptor agonist.

The baseline platelet count, broken down by TPO-RA, showed a non-normal, bimodal distribution in the ROM group, with a median baseline platelet count of 29000 (IQR, 19000-42000) for ELT treatment and 20000/µL (IQR, 14000-32000) for ROM treatment, with no statistically significant difference between the two groups (Figure 1).

Concomitant medications used during treatment are described in Table 5. 

**Table 5:** Concomitant medications during treatment with TPO-RAs. *Rescue therapies included steroids, immunoglobulin, or platelet transfusions during the treatment period. TPO-RA: thrombopoietin receptor agonist.

Variable	Overall
n	58
Anticoagulants (%)	4 (6.9)
Antiplatelet agents (%)	3 (5.2)
Steroids (%)	30 (51.7)
Azathioprine (%)	14 (24.1)
Aluminium Hydroxide (%)	1 (1.7)
Calcium or Magnesium supplements (%)	7 (12.1)
Oral contraceptives (%)	1 (1.7)
Rescue therapies* (%)	22 (37.9)

Additional thrombosis risk factors included prolonged immobilization (>3 days), which was present in 6.9% of patients. There were no reports of lower extremity fractures, major trauma, or pregnancy.

Effectiveness

The median time to platelet response was available for 31 of 37 patients in the ELT group and 15 of 21 patients in the ROM group. The median time to response was 37 days, with a 95% confidence interval (CI) of 19-61, in patients treated with ELT, and 71 days (95% CI: 12-162) in those treated with ROM. These findings show a longer time to platelet response in patients treated with ROM; however, this difference was not statistically significant (p = 0.5).

**Figure 2:**
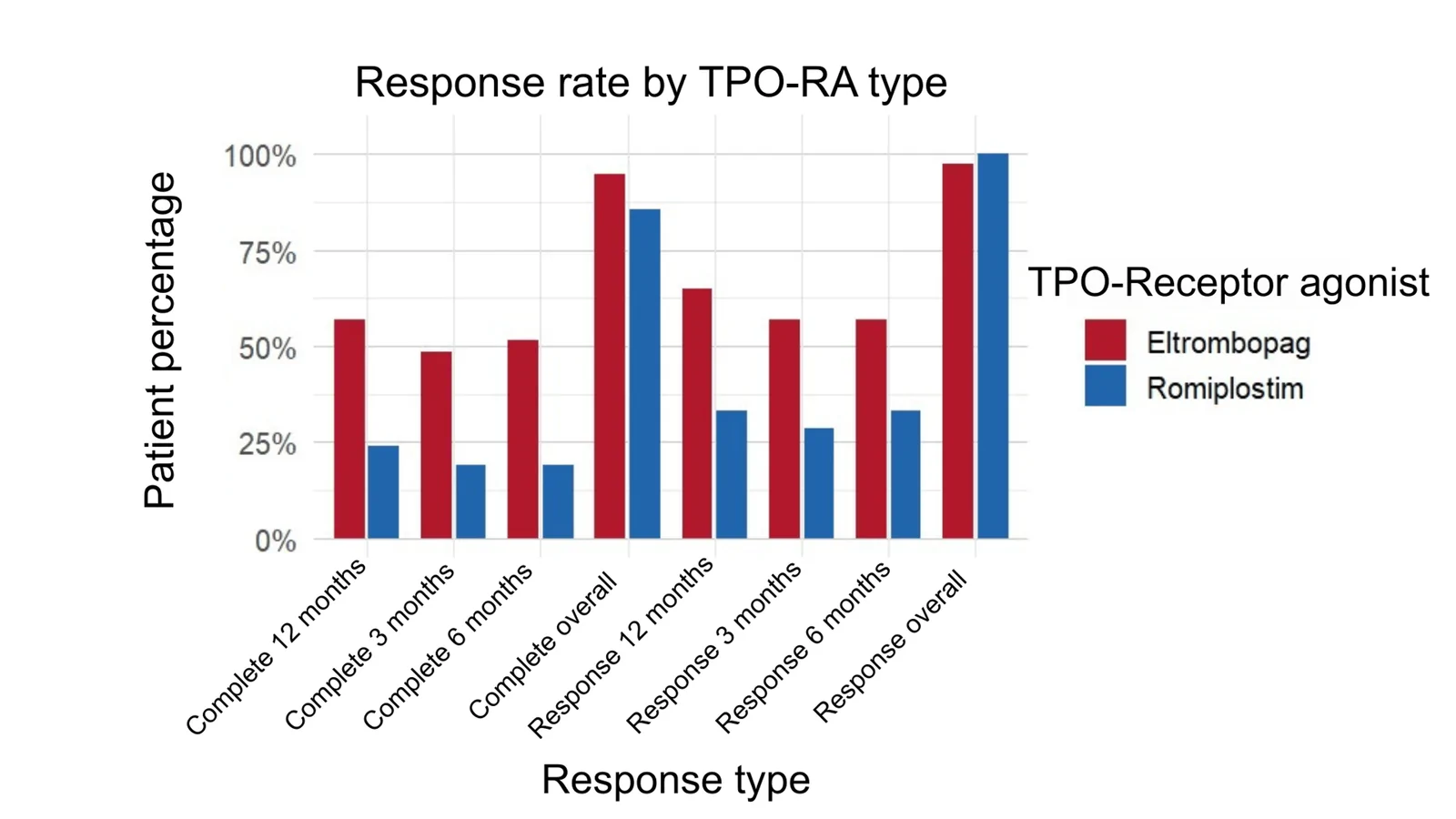
Platelet response rate at three, six, and 12 months by TPO-RA type. TPO: Thrombopoietin; TPO-RA: thrombopoietin receptor agonist. Platelet response: platelet count >30,000/µL and at least a twofold increase from the baseline platelet count in the absence of bleeding. Complete platelet response: platelet count >100,000/µL in the absence of bleeding. Definitions according to Rodeghiero et al. [4]. Figure generated using R software [20].

The proportion of patients achieving at least one platelet response according to the predefined study criteria was 97.3% for ELT and 100% for ROM (p = 1.000). The platelet response rates at three, six, and 12 months for each TPO-RA are presented in Table 6 and summarized in Figure 2. Data to evaluate this outcome were limited, particularly in the ROM group (10 of 21 patients). At three, six, and 12 months of follow-up, patients in the ELT group showed a trend toward a higher platelet response and complete platelet response; however, this could be partially attributed to the lower availability of data in the ROM group, as well as the presence of more diseased individuals with multiple prior lines of treatment in this group. These factors may also have contributed to the higher rates of steroid dependence and therapeutic failure observed in the ROM group.

**Table 6:** Platelet response at three, six, and 12 months for each TPO-RA. *The definitions of these variables correspond to the standardized definitions of Rodeghiero et al. [4]. Inf: Infinite; NA: not applicable; OR: odds ratio; TPO-RA: thrombopoietin receptor agonist.

Variable	Eltrombopag	Romiplostim	p-value	Fisher exact test value (OR)
n	37	21		
Steroid dependent (%)*	9 (24.3)	13 (61.9)	0.0102	4.9
Therapeutic failure (%)*	3 (8.1)	7 (33.3)	0.0268	5.48
Platelet response (%)*	36 (97.3)	21 (100.0)	1.000	Inf
Complete platelet response (%)*	35 (94.6)	18 (85.7)	0.341	0.350
Response at the third month (%)			0.0479	NA
Unknown	8 (21.6)	11 (52.4)		
No	8 (21.6)	4 (19.0)		
Yes	21 (56.8)	6 (28.6)		
Response at the sixth month (%)			0.217	NA
Unknown	13 (35.1)	11 (52.4)		
No	3 (8.1)	3 (14.3)		
Yes	21 (56.8)	7 (33.3)		
Response at one year (%)			0.0425	NA
Unknown	11 (29.7)	11 (52.4)		
No	2 (5.4)	3 (14.3)		
Yes	24 (64.9)	7 (33.3)		
Complete response at the third month(%)			0.0301	NA
Unknown	8 (21.6)	11 (52.4)		
No	11 (29.7)	6 (28.6)		
Yes	18 (48.6)	4 (19.0)		
Complete response at the sixth month (%)			0.0415	NA
Unknown	13 (35.1)	11 (52.4)		
No	5 (13.5)	6 (28.6)		
Yes	19 (51.4)	4 (19.0)		
Complete response at one year (%)			0.0508	NA
Unknown	11 (29.7)	11 (52.4)		
No	5 (13.5)	5 (23.8)		
Yes	21 (56.8)	5 (23.8)		

**Figure 3:**
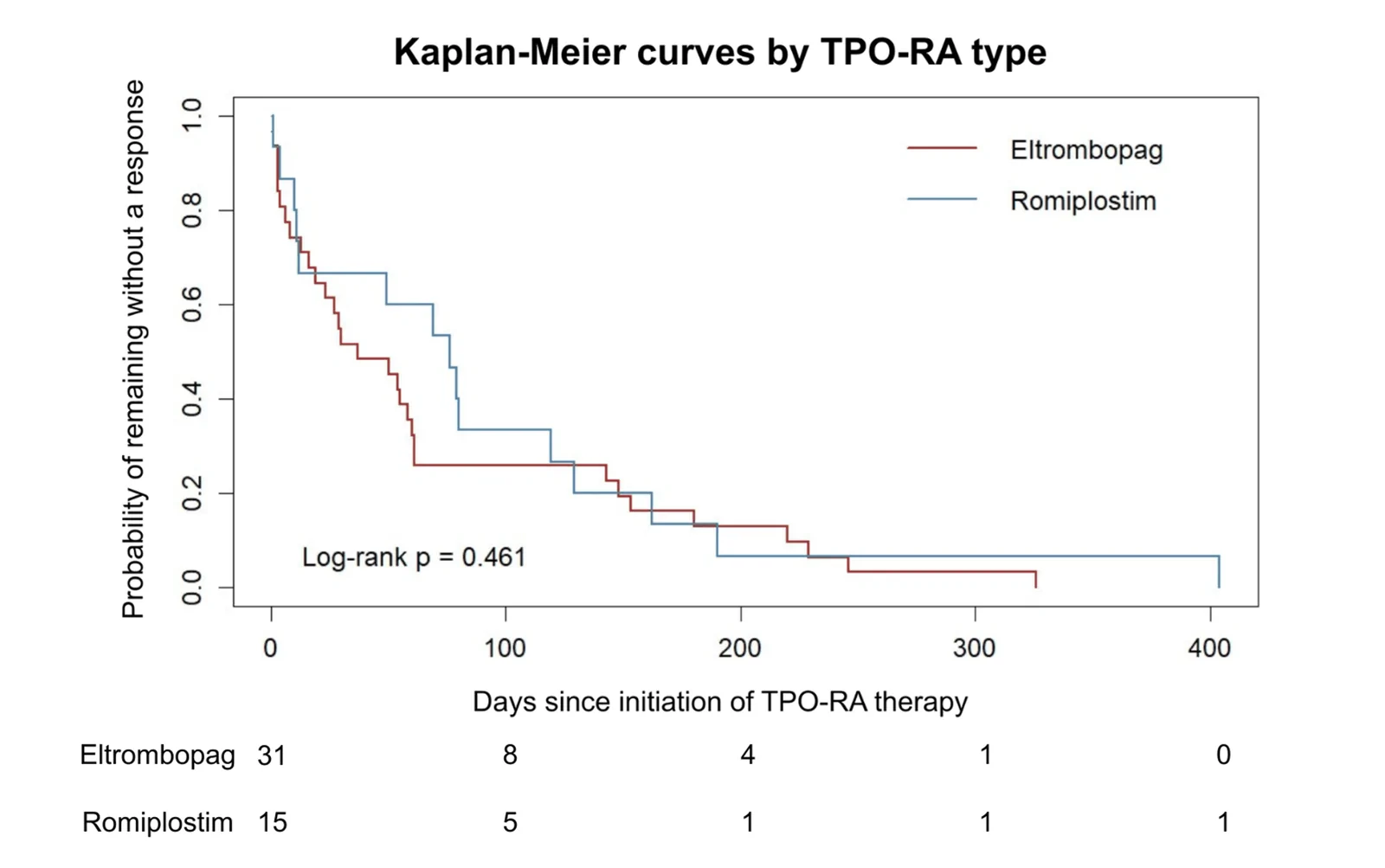
Kaplan–Meier curves for the probability of a platelet response according to TPO-RA type. TPO-RA: Thrombopoietin receptor agonist. Figure generated using R software [20].

We compared the probability of a platelet response between both groups by constructing a Kaplan-Meier survival curve. Both groups showed similar probabilities of a platelet response, with no statistically significant differences according to the log-rank test (p = 0.461; Figure 3).

Regarding the loss of platelet response, 11/37 (29.7%) patients in the ELT group and 10/21 (47.6%) in the ROM group lost their platelet response. Complete loss of platelet response was observed in 15/37 (40%) patients in the ELT group and 11/21 (52.3%) in the ROM group.

**Figure 4:**
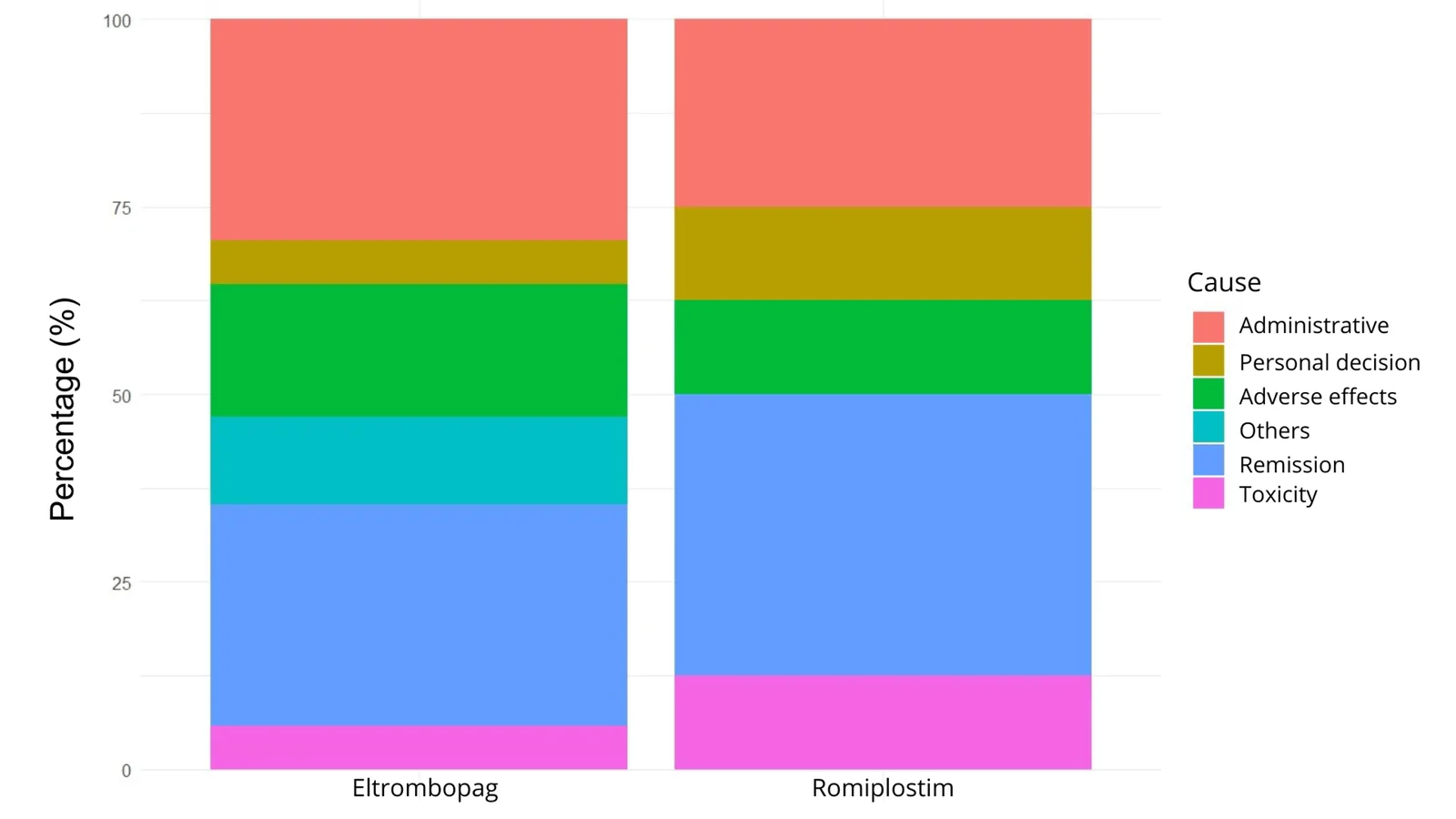
Reasons for discontinuation of each TPO-RA. Figure generated using R software [20]. TPO-RA: Thrombopoietin receptor agonist.

Treatment discontinuation was observed in 17/37 (45.9%) patients in the ELT group and 8/21 (38%) in the ROM group. Discontinuation due to remission was possible in 5/17 (29.4%) patients with ELT and 3/8 (37.5%) patients with ROM. Reasons for discontinuation also included administrative causes (drug shortages or unavailability, problems associated with the healthcare system), adverse events, hepatic or hematological toxicity, personal decisions, and other causes, as shown in Figure 4. Other causes included one patient who experienced treatment failure after switching between different brands in the ELT group, and one patient who received ELT as surgical prophylaxis and discontinued treatment after achieving the pre-surgical therapeutic target.

Safety

Complications observed during the treatment period are presented in Table 7. Because of the low frequency of adverse events, safety outcomes are presented descriptively for the overall cohort rather than stratified by treatment. Major and clinically significant bleeding events occurred in 3.4% and 17.2% of patients, respectively. Thrombotic events were reported in 8.6% of patients, with superficial venous thrombosis being the most frequent event (5.2%). Adverse events occurred in 12.1% of patients, including myelofibrosis in one patient in the ROM group and hepatotoxicity in one patient in the ELT group. Headache was the most common adverse event (6.9%), whereas nausea and arthralgia were each reported in 1.7% of patients. At the end of follow-up, all patients were alive except for one patient in the ROM group who developed myelofibrosis and refractory thrombocytopenia and died 216 days after treatment initiation. Cumulative incidence and median overall survival were not estimated because of the small number of events.

**Table 7:** Treatment-related complications associated with TPO-RAs. * Variables defined according to the World Health Organization (WHO) grading system for bleeding [19]. ^† ^Values represent the number of patients who experienced thrombotic events. A total of 11 thrombotic events were recorded, with some patients experiencing multiple events, including one patient who experienced four thrombotic events. TPO-RA: Thrombopoietin receptor agonist. ^‡ ^There were no events of lower back pain, epistaxis, cataracts, diarrhea, cough, anemia, pneumonia, acute respiratory infection, or urinary tract infection.

Variable	Overall
n	58
Mayor bleeding (%)*	2 (3.4)
Clinically significant bleeding (%)*	10 (17.2)
Thrombosis (%)^†^	5 (8.6)
Deep venous thrombosis (%)	1 (1.7)
Pulmonary embolism (%)	2 (3.4)
Acute myocardial infarction (%)	1 (1.7)
Stroke (%)	2 (3.4)
Superficial venous thrombosis (%)	3 (5.2)
Splanchnic venous thrombosis (%)	1 (1.7)
Cerebral venous thrombosis (%)	1 (1.7)
Adverse events (%)^‡^	7 (12.1)
Myelofibrosis (%)	1 (1.7)
Hepatotoxicity (%)	1 (1.7)
Headache (%)	4 (6.9)
Nausea (%)	1 (1.7)
Arthralgia (%)	1 (1.7)

## Discussion

Currently, TPO-RAs are presented as the second-line strategy of choice in the treatment of persistent or chronic ITP [7], with previous studies demonstrating their efficacy and safety [9,11]. However, head-to-head comparative studies have not been conducted, and real-world evidence remains scarce, especially in Latin American countries [22]. In this retrospective study, we identified 58 patients with primary ITP treated at a university hospital in Bogotá, Colombia. The mean age and sex distribution were similar to those reported in the international literature [23], with a mean age of 48 years and a female predominance.

The median platelet count at the start of TPO-RA treatment was 28,000/µL (IQR, 15250-32000/µL), similar to that reported in previous studies [9,11]. When stratified by TPO-RA, the baseline platelet count showed a non-normal, bimodal distribution in the ROM group and was also consistent with values reported in international cohorts [9,11]. The majority of patients (75.9%) were in the chronic phase of the disease. Notably, only 24% had undergone a previous splenectomy. Multiple studies have shown a decrease in the use of splenectomy following the introduction of TPO-RAs [24,25]. In the pivotal ROM study, only 9% of patients treated with ROM required splenectomy, compared with 36% in the standard care group, and the time to splenectomy was notably longer in the TPO-RA group [26].

Patient characteristics were similar in both groups. One exception was the number of prior therapies, with a statistically significantly higher number of patients in the ROM group (p < 0.001). This finding suggests that patients in the ROM group required multiple lines of therapy, an observation further supported by the higher proportion of patients with a history of splenectomy in this group. Differences in the number of prior therapies have been reported in some retrospective studies, attributed mainly to the earlier availability of ROM in European countries [27]. However, recent real-world studies in the United Kingdom showed no statistically significant differences in the number of prior therapies used [28]. Although subject to selection bias because of the study’s retrospective, single-center design, this finding is interesting and needs to be corroborated in future prospective studies.

The median time to platelet response was 71 days for ROM (95% CI: 12-162 days). A pivotal ROM study reported a median time to platelet response of 2-3 weeks [8], while observational studies have reported median times to a platelet response of around seven days [29]. This difference could be partly attributed to the different definitions of platelet response used in previous studies, which defined it as the first time platelet counts >50,000/µL were achieved, while we adhered to the standardized definitions of Rodeghiero et al. [4], which require platelet counts >30,000/µL and at least double the initial platelet count to be considered a platelet response. The median time to a platelet response for ELT was 37 days (95% CI: 19-61 days), which is similar to that reported in the literature [12,30]. Survival analysis using Kaplan-Meier curves comparing the probability of a platelet response for both groups showed no statistically significant differences, confirming the findings of international studies in our study population [22,29].

The overall platelet response rate was 97.3% for ELT and 100% for ROM. Insufficient data precluded adequate comparison of platelet responses at three, six, and 12 months, given that more than half of ROM-treated patients had missing follow-up platelet response data. Rates of treatment failure and steroid dependence were higher in the ROM group; however, these differences likely reflect the greater proportion of patients with more advanced disease who had received multiple prior lines of therapy, as well as the limited availability of follow-up data. With respect to treatment discontinuation, comparable rates of discontinuation due to remission, administrative issues, patient preference, and adverse events were observed in both TPO-RA groups. Safety outcomes, including thrombotic and treatment-related adverse events, were infrequent and broadly consistent with rates reported in prior clinical trials and real-world studies [8,12,28].

This study has several limitations. First, its retrospective, single-center design is susceptible to selection bias and confounding by indication. In addition, missing outcome data, particularly in the romiplostim (ROM) group at the three-, six-, and 12-month follow-up assessments, may have influenced the comparative analyses, as the statistical comparisons may partially reflect differences in missingness between treatment groups rather than true differences in treatment response. The retrospective design also limited our ability to fully characterize treatment exposure because information on treatment duration, starting doses, maximum doses, and dose titration was not consistently available in the electronic health records. Furthermore, treatment selection was not protocolized during the study period and was based on the treating hematologist's clinical judgment. Because the rationale for selecting ROM or ELT was not consistently documented in the medical records, the factors underlying the observed baseline differences between groups could not be determined retrospectively, and residual confounding by indication cannot be excluded. The exclusion of patients with less than three months of follow-up, a criterion established a priori to ensure adequate assessment of study outcomes, may also have introduced survivorship bias, potentially inflating the observed response rates. Finally, the relatively small sample size precluded multivariable analyses to adjust for baseline differences between treatment groups and limited the statistical power to detect clinically meaningful differences. Accordingly, the comparative findings should be interpreted cautiously and regarded as exploratory and hypothesis-generating, pending confirmation in larger prospective studies.

To the authors’ knowledge, this study is the first real-world retrospective study on the effectiveness and safety of ROM and ELT conducted in Colombia. Its strengths include the description of effectiveness and safety variables, the long-term follow-up enabled by the retrospective design, and the description of the population characteristics of each treatment group, with data corroborated by the literature and suggesting a more severely compromised population at the start of treatment in the ROM group.

## Conclusions

In this real-world retrospective study, no statistically significant differences were observed between ROM and ELT in the probability of platelet response or time to platelet response. Safety outcomes, including thrombotic events and treatment-related adverse events, were infrequent in both treatment groups. Patients treated with ROM had received more prior therapies and more frequently had a history of splenectomy, suggesting a population with more advanced disease at treatment initiation. These findings provide real-world comparative data on TPO-RAs from a Latin American university hospital and should be interpreted as exploratory and hypothesis-generating. Larger prospective studies are needed to confirm these observations.

## Appendices

**Table 8:** STROBE checklist *Give information separately for cases and controls in case-control studies and, if applicable, for exposed and unexposed groups in cohort and cross-sectional studies.

	Item No.	Recommendation	Page No.	Relevant text from manuscript
Title and abstract	1	(a) Indicate the study’s design with a commonly used term in the title or the abstract	1	Title: Effectiveness and Safety of Two Thrombopoietin Receptor Agonists: A Retrospective Cohort Study of Adults With Primary Immune Thrombocytopenia. Abstract (Methodology): A retrospective cohort study was conducted using medical records of adults with primary ITP treated at the Hospital Universitario Nacional de Colombia (Colombia National University Hospital) between 2016 and 2023.
		(b) Provide in the abstract an informative and balanced summary of what was done and what was found	1	Methodology: A retrospective cohort study was conducted using medical records of adults with primary ITP treated at the Hospital Universitario Nacional de Colombia (Colombia National University Hospital) between 2016 and 2023. Effectiveness and safety outcomes are described, and the probability of platelet response was determined using Kaplan-Meier analysis. Results: A total of 58 patients were included (63.8% women), with a mean age of 48 years. The median number of prior treatments was 3.00 (interquartile range [IQR], 3.00-4.00) for the ROM group and 2.00 (IQR, 2.00-3.00) for the ELT group (p < 0.001). The median time to achievement of the first platelet response was 37 days (95% confidence interval [CI], 19-61) in patients treated with ELT and 71 days (95% CI, 12-162) in those treated with ROM (p = 0.50). The proportion of patients achieving at least one platelet response during follow-up was 97.3% for ELT and 100% for ROM (p = 1.000). No statistically significant difference in the probability of achieving a platelet response was observed between treatment groups (log-rank p = 0.461). Thrombotic events and treatment-related adverse events were infrequent.
Introduction				
Background/rationale	2	Explain the scientific background and rationale for the investigation being reported	1, 2	Primary immune thrombocytopenia (ITP) is a disease characterized by immune system-mediated platelet destruction and decreased platelet production. Although the exact pathophysiological mechanisms involved are not fully understood, proposed mechanisms include the development of autoantibodies directed against platelet antigens resulting in their destruction in the reticuloendothelial system, as well as damage to megakaryocytes in the bone marrow and impaired platelet production. Primary ITP is estimated to have an annual incidence of approximately 3/100,000 people, with peaks observed in young women and older adults. The development of TPO-RAs as second-line drugs has led to a greater response to treatment and a reduction in the need for splenectomy or immunosuppression. Although studies have demonstrated the efficacy and safety of TPO-RAs in adults, including some meta-analyses of indirect comparisons, head-to-head comparisons between these drugs are lacking. Consequently, comparative evidence for outcomes such as time to platelet response, probability of platelet response, clinically significant bleeding events, or thrombotic events remains limited.
Objectives	3	State specific objectives, including any prespecified hypotheses	2	describe the effectiveness and safety of ROM and ELT in adults with primary ITP treated at a Colombian university hospital and to explore comparative outcomes between treatment groups.
Methods				
Study design	4	Present key elements of study design early in the paper	1	Methodology: A retrospective cohort study was conducted using medical records of adults with primary ITP treated at the Hospital Universitario Nacional de Colombia (Colombia National University Hospital) between 2016 and 2023. Effectiveness and safety outcomes are described, and the probability of platelet response was determined using Kaplan-Meier analysis.
Setting	5	Describe the setting, locations, and relevant dates, including periods of recruitment, exposure, follow-up, and data collection	2	We present a real-world retrospective cohort study conducted at a teaching hospital in Bogotá, Colombia. A review of electronic health records was performed for all adults diagnosed with primary ITP and treated with ROM or ELT at the Hospital Universitario Nacional de Colombia (Colombia National University Hospital), between January 2016 and June 2023. The exposure was defined as treatment with either ELT or ROM. Follow-up was defined as the interval from initiation of TPO-RA therapy to the date of the last available patient visit recorded in the medical record.
Participants	6	(a) Cohort study—Give the eligibility criteria, and the sources and methods of selection of participants. Describe methods of follow-up Case-control study—Give the eligibility criteria, and the sources and methods of case ascertainment and control selection. Give the rationale for the choice of cases and controls Cross-sectional study—Give the eligibility criteria, and the sources and methods of selection of participants	2	Patient selection Electronic health records were filtered to select patients according to ICD-10 classification] as D693 Idiopathic Thrombocytopenic Purpura, D694 Other Primary Thrombocytopenias, or D473 Thrombocytopenia (Essential Hemorrhage), with further review to include only adult patients with primary ITP and treatment with either ROM or ELT between January 1, 2016, and June 30, 2023. Exclusion criteria included secondary immune thrombocytopenia, aplastic anemia, and hereditary thrombocytopenias or congenital bone marrow failure syndromes. Data extraction was performed using a specific data collection form in the REDCap platform. Data were extracted independently with revision by two authors, and any disagreement was resolved by consensus or with the help of a third author.
		(b) Cohort study—For matched studies, give matching criteria and number of exposed and unexposed Case-control study—For matched studies, give matching criteria and the number of controls per case		Not applicable (not a matched study)
Variables	7	Clearly define all outcomes, exposures, predictors, potential confounders, and effect modifiers. Give diagnostic criteria, if applicable	3	The exposure was defined as treatment with either ELT or ROM. Follow-up was defined as the interval from initiation of TPO-RA therapy to the date of the last available patient visit recorded in the medical record. TPO-RA doses were initiated and adjusted at the discretion of the treating hematologist according to platelet response and institutional practice, which was based on international ITP guidelines. Effectiveness outcomes included time to the first platelet response; platelet response and complete platelet response at 3, 6, and 12 months after treatment initiation; therapeutic failure; and steroid dependence. Platelet response, complete platelet response, therapeutic failure, and steroid dependence were defined according to the standardized criteria proposed by Rodeghiero et al. [4]. The number of patients who discontinued treatment because of remission (platelet count >100,000/µL for >1 year) was also recorded for each TPO-RA. Regarding treatment safety, thrombotic events and adverse events, including hepatotoxicity and myelofibrosis, were evaluated. The number of major bleeding events, defined by the World Health Organization (WHO) grading system for bleeding [19] as WHO grades 3-4, and clinically significant bleeding events (WHO grade ≥2) was assessed.
Data sources/ measurement	8*	For each variable of interest, give sources of data and details of methods of assessment (measurement). Describe comparability of assessment methods if there is more than one group	2, 3	A review of electronic health records was performed for all adults diagnosed with primary ITP and treated with ROM or ELT at the Hospital Universitario Nacional de Colombia (Colombia National University Hospital), between January 2016 and June 2023. The study was approved by the ethics committee of the hospital, the approval is recorded in the minutes of the meeting No. CEI-HUN-ACTA-2023-12 (Project ID: CEI-2023-10-02). Data extraction was performed using a specific data collection form in the REDCap platform. These variables were defined according to the standardized definitions of Rodeghiero et al. Data were extracted independently with revision by two authors, and any disagreement was resolved by consensus or with the help of a third author.
Bias	9	Describe any efforts to address potential sources of bias	3	Data were extracted independently with revision by two authors, and any disagreement was resolved by consensus or with the help of a third author. Platelet response, complete platelet response, therapeutic failure, and steroid dependence were defined according to the standardized criteria proposed by Rodeghiero et al. [4]. all eligible patients meeting the inclusion criteria during the study period were included.
Study size	10	Explain how the study size was arrived at	3	No a priori sample size or statistical power calculation was performed because all eligible patients meeting the inclusion criteria during the study period were included.

Quantitative variables	11	Explain how quantitative variables were handled in the analyses. If applicable, describe which groupings were chosen and why	3	A descriptive statistical analysis was performed, including medians, interquartile ranges (IQRs), and percentages. Since the data obtained showed a non-normal distribution, non-parametric methods were used for their analysis, with differences in categorical variables analyzed using the Fisher exact test, and differences in continuous variables using the Mann-Whitney U test. Kaplan-Meier curves were constructed for the platelet response probability survival analysis, with calculation of the log-rank test.
Statistical methods	12	(a) Describe all statistical methods, including those used to control for confounding	3	A descriptive statistical analysis was performed, including medians, interquartile ranges (IQRs), and percentages. Since the data obtained showed a non-normal distribution, non-parametric methods were used for their analysis, with differences in categorical variables analyzed using the Fisher exact test, and differences in continuous variables using the Mann-Whitney U test. Kaplan-Meier curves were constructed for the platelet response probability survival analysis, with calculation of the log-rank test. Time zero for the Kaplan-Meier analysis was defined as the date of initiation of TPO-RA therapy. The event was the achievement of platelet response according to the predefined study criteria. Patients with missing dates of platelet response were excluded from the time-to-event analysis because the event time could not be determined. Patients who did not achieve a platelet response were treated as censored at their last available follow-up. No multivariable adjustment or other methods to control for confounding were performed because of the limited sample size and exploratory nature of the study.
		(b) Describe any methods used to examine subgroups and interactions		No subgroup analysis were done
		(c) Explain how missing data were addressed	3	Missing data were not imputed. For the analyses of platelet response at 3, 6, and 12 months, patients with unavailable response data were retained as an "Unknown" category in the contingency tables.
		(d) Cohort study—If applicable, explain how loss to follow-up was addressed Case-control study—If applicable, explain how matching of cases and controls was addressed Cross-sectional study—If applicable, describe analytical methods taking account of sampling strategy	3	Follow-up was defined as the interval from initiation of TPO-RA therapy to the date of the last available patient visit recorded in the medical record. Patients with less than 3 months of follow-up were excluded according to the predefined eligibility criteria.
		(e) Describe any sensitivity analyses		No sensitivity analyses were performed.
Results				
Participants	13*	(a) Report numbers of individuals at each stage of study—eg numbers potentially eligible, examined for eligibility, confirmed eligible, included in the study, completing follow-up, and analysed	3	A total of 112 patients who met the inclusion criteria were identified. 54 patients were excluded for the following reasons: 12 due to insufficient data, 33 with a follow-up time of less than 3 months, 2 who received treatment outside the follow-up period, and 5 due to concomitant treatment with ROM and ELT. Among the 58 included patients, 63.8% were women and the mean age was 48 years. Fewer than half had prior comorbidities.
		(b) Give reasons for non-participation at each stage	3	54 patients were excluded for the following reasons: 12 due to insufficient data, 33 with a follow-up time of less than 3 months, 2 who received treatment outside the follow-up period, and 5 due to concomitant treatment with ROM and ELT.
		(c) Consider use of a flow diagram		No flow diagram was created
Descriptive data	14*	(a) Give characteristics of study participants (eg demographic, clinical, social) and information on exposures and potential confounders	4, 5	Table 1: Population comorbidities. Table 2: General characteristics of the study population.
		(b) Indicate number of participants with missing data for each variable of interest	8	Table 6: Platelet response at 3, 6, and 12 months for each TPO-RA. Patients reported under the unknown category. The median time to platelet response was available for 31 of 37 patients in the ELT group and 15 of 21 patients in the ROM group.
		(c) Cohort study—Summarise follow-up time (eg, average and total amount)	5	Table 3 Median follow-up time, days (IQR). ELT: 716.0 (287.0–1186.0); ROM: 955.0 (567.0–1451.0).
Outcome data	15*	Cohort study—Report numbers of outcome events or summary measures over time	8, 11	Table 6: Reports response and complete response rates at 3, 6, and 12 months. Table 7: Reports the number and percentage of major bleeding events (3.4%), clinically significant bleeding (17.2%), thrombosis (8.6%), and adverse events (12.1%).
		Case-control study—Report numbers in each exposure category, or summary measures of exposure		
		Cross-sectional study—Report numbers of outcome events or summary measures		
Main results	16	(a) Give unadjusted estimates and, if applicable, confounder-adjusted estimates and their precision (eg, 95% confidence interval). Make clear which confounders were adjusted for and why they were included	7 - 11	Effectiveness The median time to platelet response was available for 31 of 37 patients in the ELT group and 15 of 21 patients in the ROM group. The median time to response was 37 days, with a 95% confidence interval (CI) of 19-61, in patients treated with ELT, and 71 days (95% CI: 12-162) in those treated with ROM. These findings show a longer time to platelet response in patients treated with ROM; however, this difference was not statistically significant (p = 0.5). The proportion of patients achieving at least one platelet response according to the predefined study criteria was 97.3% for ELT and 100% for ROM (p = 1.000). The platelet response rates at 3, 7 of 146, and 12 months for each TPO-RA are presented in Table 6 and summarized in Figure 2. Data to evaluate this outcome were limited, particularly in the ROM group (10 of 21 patients). Safety Complications observed during the treatment period are presented in Table 7. Because of the low frequency of adverse events, safety outcomes are presented descriptively for the overall cohort rather than stratified by treatment. Major and clinically significant bleeding events occurred in 3.4% and 17.2% of patients, respectively. Thrombotic events were reported in 8.6% of patients, with superficial venous thrombosis being the most frequent event (5.2%). Adverse events occurred in 12.1% of patients, including myelofibrosis in one patient in the ROM group and hepatotoxicity in one patient in the ELT group. Headache was the most common adverse event (6.9%), whereas nausea and arthralgia were each reported in 1.7% of patients. At the end of follow-up, all patients were alive except for one patient in the ROM group who developed myelofibrosis and refractory thrombocytopenia and died 216 days after treatment initiation.
		(b) Report category boundaries when continuous variables were categorized	4	Table 1: The Charlson comorbidity index was categorized into the ranges of 0, 1-2, 3-6 and >7.
		(c) If relevant, consider translating estimates of relative risk into absolute risk for a meaningful time period		Not applicable (relative risks and incidence rate ratios were not calculated because of the low frequency of outcome events and the exploratory nature of the study).

Other analyses	17	Report other analyses done—eg analyses of subgroups and interactions, and sensitivity analyses		No other analysis done
Discussion				
Key results	18	Summarise key results with reference to study objectives	11	In this retrospective study, we identified 58 patients with primary ITP treated at a university hospital in Bogotá, Colombia. The mean age and sex distribution were similar to those reported in the international literature, with a mean age of 48 years and a female predominance. The median platelet count at the start of TPO-RA treatment was 28,000/μL (range: 2,000-100,000/μL),similar to that reported in previous studies. When stratified by TPO-RA, the baseline platelet count showed a non-normal, bimodal distribution in the ROM group and was also consistent with values reported in international cohorts. The majority of patients (75.9%) were in the chronic phase of the disease. Patient characteristics were similar in both groups. One exception was the number of prior therapies, with a statistically significantly higher number of patients in the ROM group (p < 0.001). This finding suggests that patients in the ROM group required multiple lines of therapy, an observation further supported by the higher proportion of patients with a history of splenectomy in this group.
Limitations	19	Discuss limitations of the study, taking into account sources of potential bias or imprecision. Discuss both direction and magnitude of any potential bias	12	This study has several limitations. First, its retrospective, single-center design is susceptible to selection bias and confounding by indication. In addition, missing outcome data, particularly in the romiplostim (ROM) group at the 3-, 6-, and 12-month follow-up assessments, may have influenced the comparative analyses, as the statistical comparisons may partially reflect differences in missingness between treatment groups rather than true differences in treatment response. The retrospective design also limited our ability to fully characterize treatment exposure because information on treatment duration, starting doses, maximum doses, and dose titration was not consistently available in the electronic health records. Furthermore, treatment selection was not protocolized during the study period and was based on the treating hematologist's clinical judgment. Because the rationale for selecting ROM or ELT was not consistently documented in the medical records, the factors underlying the observed baseline differences between groups could not be determined retrospectively, and residual confounding by indication cannot be excluded. The exclusion of patients with less than three months of follow-up, a criterion established a priori to ensure adequate assessment of study outcomes, may also have introduced survivorship bias, potentially inflating the observed response rates. Finally, the relatively small sample size precluded multivariable analyses to adjust for baseline differences between treatment groups and limited the statistical power to detect clinically meaningful differences. Accordingly, the comparative findings should be interpreted cautiously and regarded as exploratory and hypothesis-generating, pending confirmation in larger prospective studies.
Interpretation	20	Give a cautious overall interpretation of results considering objectives, limitations, multiplicity of analyses, results from similar studies, and other relevant evidence	12	In this real-world retrospective study, no statistically significant differences were observed between ROM and ELT in the probability of platelet response or time to platelet response. Safety outcomes, including thrombotic events and treatment-related adverse events, were infrequent in both treatment groups. Patients treated with ROM had received more prior therapies and more frequently had a history of splenectomy, suggesting a population with more advanced disease at treatment initiation. These findings provide real-world comparative data on TPO-RAs from a Latin American university hospital and should be interpreted as exploratory and hypothesis-generating. Larger prospective studies are needed to confirm these observations.
Generalisability	21	Discuss the generalisability (external validity) of the study results	12	These findings provide real-world comparative data on TPO-RAs from a Latin American university hospital and should be interpreted as exploratory and hypothesis-generating. The findings may be generalizable to adults with primary ITP treated in similar tertiary-care hospitals in Latin America. However, generalizability is limited by the single-center design and the relatively small sample size.
Other information				
Funding	22	Give the source of funding and the role of the funders for the present study and, if applicable, for the original study on which the present article is based	13	Payment/services info: All authors have declared that no financial support was received from any organization for the submitted work. Financial relationships: All authors have declared that they have no financial relationships at present or within the previous three years with any organizations that might have an interest in the submitted work. Other relationships: All authors have declared that there
